# Supplementary material for: Reducing the Periplasmic Glutathione Content Makes Escherichia coli Resistant to Trimethoprim and Other Antimicrobial Drugs
Source: Microbiol Spectr. 2021 Dec 15;9(3):e00743-21. doi: 10.1128/Spectrum.00743-21 (PMC8672908; doi:10.1128/Spectrum.00743-21)
Supplement: SUPPLEMENTAL FILE 1 — Supplemental material. Download SPECTRUM00743-21_Supp_1_seq4.pdf, PDF file, 0.3 MB [file spectrum00743-21_supp_1_seq4.pdf]

Supplemental Materials

FILE S1 Quantification of intracellular TMP via LC-MS.

TMP analysis was detected using an HPLC system (Ultimate 3000 HPLC, Thermo Fisher, CA, USA) coupled to an AB Sciex 4500 triple-quadruple mass spectrometer (LC-MS/MS). Samples were filtered and centrifuged at 10,000 g for 5 min, and these supernatants were further diluted with purified water (1:1, v/v). The injector temperature was set to 20°C, and the column was maintained at 40°C. The separation was performed on a column Syncronis C18 (1.7 μm, 2.1 mm × 100 mm, Thermo Fisher) after injection of a 5 μL sample aliquot. The gradient elution, with 0.1% (v/v) formic acid in methanol (A) and 0.1% (v/v) formic acid in water (B) at a flow rate of 0.3 mL/min, was optimized as follows: 0–5 min (90–30% B), hold for 3 min (30% B), 8.01 min (90% B), and equilibrate for 2 min (90% B). The MS/MS was operated in the multiple reaction monitoring (MRM) mode. The LC-MS/MS parameters of TMP are provided in Table S8. The retention time of TMP was 5.32 min. Quantification was conducted using a standard calibration curve consisting of a concentration gradient (0.1, 0.2, 0.5, 1.0, 2.0, 5.0, and 10 ng mL^{-1}) with the deviation of every point from the regression line being less than 20% from that of its theoretical value. The regression coefficients (r^2) of the calibration curve for the target analyte were higher than 0.99. The TMP concentration was finally normalized to the OD600, the reading of which was taken from the original bacterial culture.

TABLE S1 TMP MICs in Enterohemorrhagic *E. coli* (EHEC) and *E. coli* BW25113 and MG1655 strains.

Strains	MIC for TMP (μg/mL)	Fold change
EHEC O157:H7	0.64	–
EHEC O157:H7::*Kan*	0.64	–
EHEC O157:H7Δ*gshA::Kan*	2.56	4
EHEC O157:H7Δ*gshB::Kan*	2.56	4
EHEC O157:H7Δ*grxA*	1.28	2
EHEC O157:H7Δ*cydD::Kan*	5.12	8
E. coli ATCC 25922	2.56	–
E. coli ATCC 25922 Δ*gshA*	20.48	8
E. coli ATCC 25922 Δ*gshB*	10.24	4
E. coli ATCC 25922 Δ*grxA*	5.12	2
E. coli ATCC 25922 Δ*cydD*	10.24	4
E. coli BW25113	0.32	–
E. coli BW25113 Δ*gshA*	1.28	4
E. coli BW25113 Δ*gshB*	1.28	4
E. coli BW25113 Δ*grxA*	0.64	2
E. coli BW25113 Δ*cydD*	1.28	4
E. coli MG1655	0.32	–
E. coli MG1655 Δ*gshA*	1.28	4
E. coli MG1655 Δ*gshB*	1.28	4
E. coli MG1655 Δ*grxA*	0.32	–
E. coli MG1655 Δ*cydD*	1.28	4

TABLE S2 Susceptibilities of E. coli W3110 Δ*cydD* and complement strain to multiple antimicrobial drugs with different IPTG concentration.

Strains	IPTG(μm)	MIC[a] (μg/mL) of							
		TMP	K	Neo	Gen	Spe	SM	Cm	RIF
E. coli W3110		0.32	5	5	2	20	8	5	8
E. coli W3110 Δ*cydD*	0	1.28	40	40	16	40	32	10	16
E. coli W3110 Δ*cydD* pCA24N::*cydD*		0.32	10	10	8	40	16	—	16
E. coli W3110		0.32	5	5	2	20	8	5	8
E. coli W3110 Δ*cydD*	2.5	1.28	40	40	16	40	32	10	16
E. coli W3110 Δ*cydD* pCA24N::*cydD*		0.32	10	10	8	40	16	—	16
E. coli W3110		0.32	5	5	2	20	8	5	8
E. coli W3110 Δ*cydD*	5	1.28	40	40	16	40	32	10	16
E. coli W3110 Δ*cydD* pCA24N::*cydD*		0.32	20	20	8	40	16	—	16

[a]TMP, trimethoprim; K, kanamycin; Neo, neomycin; Gen, gentamicin; Spe, spectinomycin; SM, streptomycin; Cm, chloramphenicol; RIF, rifampin.

TABLE S3 Comparative analysis for target gene mutations in NCBI pathogen list.

TABLE S4 GSH content in *E. coli* W3110, Δ*grxA*, Δ*cydD,* and Δ*gshA*.

Strains	GSH_{in} (µm/OD_{600})	GSH_{per} (µm/OD_{600})
E. coli W3110	24.32 ± 0.81 (1.0)	9.98 ± 0.44 (1.0)
E. coli W3110 Δ*grxA*	26.49 ± 0.89 (1.09)	3.98 ± 0.52 (0.4)
E. coli W3110 Δ*cydD*	23.00 ± 0.98 (0.95)	6.31 ± 0.80 (0.63)
E. coli W3110 Δ*gshA*	0	0

Data are presented the mean ± SD and set as the ratio between the values in mutants and wild-type strain in parentheses.

TABLE S5 The effects of exogenous GSH treatments on TMP sensitivity in different strains.

Strains	MIC for TMP (mg/L)	
	No addition	2 mM GSH
E. coli W3110	0.32	0.32
E. coli W3110 Δ*cydD*	1.28	0.32
E. coli W3110 Δ*grxA*	1.28	0.64
E. coli W3110 Δ*gshA*	1.28	0.64
E. coli W3110 Δ*gshB*	1.28	0.64

TABLE S6 The gene expression levels of *acrAB*, *tolC*, and *soxS* in different strains.

Strains	*acrA*[a]		*acrB*		*tolC*		*soxS*	
	FC[b]	R[c]	FC[b]	R[c]	FC[b]	R[c]	FC[b]	R[c]
E. coli W3110	1	–	1	–	1	–	1	–
E. coli W3110 Δ*grxA*	3.20	UP	6.29	UP	2.28	UP	5.05	UP
E. coli W3110 Δ*cydD*	1.55	UP	2.69	UP	2.97	UP	3.12	UP
E. coli W3110 Δ*cpxR*	0.72	Down	0.71	Down	0.63	Down	0.71	Down
E. coli W3110 Δ*cydD*Δ*cpxR*	0.56	Down	0.51	Down	0.31	Down	0.37	Down

X[a] means the Different gene names; FC[b] means the fold change of gene expression ($2^{-\Delta\Delta Ct}$) which normalized by the *E. coli* W3110; R[c] means the regulation of gene expression which was up-regulated or down-regulated.

TABLE S7 The Relative concentration levels of TMP accumulated within different *E. coli* strains.

Strains	TMP (ng/ml/OD_{600})
E. coli W3110	7.48 ± 0.19 (1.0)
E. coli W3110 Δ*cydD*	4.87 ± 0.17 (0.65)
E. coli W3110 Δ*cydD*Δ*acrA*	8.09 ± 0.03 (1.08)
E. coli W3110 Δ*cydD*Δ*tolC*	8.22 ± 0.25 (1.10)
E. coli W3110 Δ*acrA*	8.93 ± 0.05 (1.19)
E. coli W3110 Δ*acrB*	9.06 ± 0.30 (1.21)
E. coli W3110 Δ*tolC*	9.02 ± 0.06 (1.21)

Data are presented the mean ± SD and set as the ratio between the values in mutants and wild-type strain in parentheses.

TABLE S8 LC-MS/MS parameters of individual TMP

Compound	Q1 (m/z)	Q3 (m/z)	DP (V)	EP (V)	CE (ev)	CXP (V)
TMP	291.000	261.000	110.0	4.5	32.0	12.0
	291.000	123.000	110.0	4.5	32.0	9.0

TABLE S9 Plasmids, strains and primers used in this study.

Category	Name	Relevant Features/Sequences	Source
Strains			
	E. coli W3110		Lab stock
	E. coli W3110 Δ*X*	*X* gene was deleted in *E. coli* W3110	This study
	E. coli W3110 Δ*cydD*Δ*X*	*X* gene was deleted in *E. coli* W3110 Δ*cydD*	This study
	E. coli W3110 Δ*grxA*Δ*X*	*X* gene was deleted in *E. coli* W3110 Δ*grxA*	This study
	E. coli W3110 Δ*gshA*Δ*X*	*X* gene was deleted in *E. coli* W3110 Δ*gshA*	This study
	E. coli W3110 Δ*gshB*Δ*X*	*X* gene was deleted in *E. coli* W3110 Δ*gshB*	This study
	E. coli BW25113		Lab stock
	E. coli BW25113 Δ*X*	*X* gene was deleted in *E. coli* BW25113	This study
	E. coli MG1655		Lab stock
	E. coli MG1655 Δ*X*	*X* gene was deleted in *E. coli* MG1655	This study
Primers			
	gshA ko-F	5-TTGATCCCGGACGTATCACAGGCGCTGGCCTGGCTGGAAAAACATCCTCAATATGAATATCCTCCTTAG-3	This study
	gshA ko-R	5-TCAGGCGTGTTTTTCCAGCCACACCGCAAACGGTTCGGTATCAGCGGCTTTGTAGGCTGGAGCTGCTTCG-3	This study
	gshB ko-F	5-ATGATCAAGCTCGGCATCGTGATGGACCCCATCGCAAACATCAACATCAAATATGAATATCCTCCTTAG-3	This study
	gshB ko-R	5-TTACTGCTGCTGTAAACGTGCTTCGATGGCATCCATTAACATTCCGGTGATGTAGGCTGGAGCTGCTTCG-3	This study
	grxA ko-F	5-ATGCAAACCGTTATTTTTGGTCGTTCGGGTTGCCCTTACTGTGTGCGTGCATATGAATATCCTCCTTAG-3	This study
	grxA ko-R	5-TCAGGCGTCCAGATTTTCTTTCACCCATGCAGCAAAATCGGTATAGCCGCTGTAGGCTGGAGCTGCTTCG-3	This study
	cydD ko-F	5-ATGAATAAATCTCGTCAAAAAGAGTTAACCCGCTGGTTAAAACAGCAAAGATATGAATATCCTCCTTAG-3	This study
	cydD ko-R	5-TTAAATCTCCTCCTGACGATGGGCCAGTAATGTGGCGAATGGGCCACCAGTGTAGGCTGGAGCTGCTTCG-3	This study
	gsiA ko-F	5-TTGCCACACAGTGATGAACTTGATGCCGGTAATGTGCTGGCGGTTGAAAAATATGAATATCCTCCTTAG-3	This study
	gsiA ko-R	5-TTATCTACGCATGAATGCGTATTCTGATTGTGGTTGTGCGACGTAATGCCTGTAGGCTGGAGCTGCTTCG-3	This study
	gsiB ko-F	5-ATGGCAAGAGCTGTACACCGTAGTGGGTTAGTGGCGCTGGGCATTGCGACATATGAATATCCTCCTTAG-3	This study
	gsiB ko-R	5-TTATTGCAAATCCGCGTCTTCAAAGCTGAAGCCGGTGTCTGGCATGATCCTGTAGGCTGGAGCTGCTTCG-3	This study
	gsiAB ko-F	5-TTGCCACACAGTGATGAACTTGATGCCGGTAATGTGCTGGCGGTTGAAAAATATGAATATCCTCCTTAG-3	This study
	gsiAB ko-R	5-TTATTGCAAATCCGCGTCTTCAAAGCTGAAGCCGGTGTCTGGCATGATCCTGTAGGCTGGAGCTGCTTCG-3	This study
	gsiC ko-F	5-ATGCTTAATTACGTTATCAAACGCTTACTGGGGTTGATTCCGACGCTGTTATATGAATATCCTCCTTAG-3	This study
	gsiC ko-R	5-TTACTTGTACCTGATAGCCGGGTTAATGGCGGCGTAAAGCACATCCACCATGTAGGCTGGAGCTGCTTCG-3	This study
	gsiD ko-F	5-ATGCGACTATTTAACTGGCGACGTCAGGCGGTGTTAAACGCCATGCCACTATATGAATATCCTCCTTAG-3	This study
	gsiD ko-R	5-CTATCCTTTAATTTTCGGATCCAGCGCATCGCGTAAACCATCGCCCAACATGTAGGCTGGAGCTGCTTCG-3	This study

ggt ko-F	5-ATGATAAAACCGACGTTTTTACGCCGGGTGGCCATTGCTGCTCTGCTCTCATATGAATATCCTCCTTAG-3	This study
ggt ko-R	5-TTAGTACCCCGCCGTTAAATCATCCACCGAGCGCGGGTCGGATGCGCCGTTGTAGGCTGGAGCTGCTTCG-3	This study
acrA ko-F	5-ATGAACAAAAACAGAGGGTTTACGCCTCTGGCGGTCGTTCTGATGCTCTCATATGAATATCCTCCTTAG-3	This study
acrA ko-R	5-TTAAGACTTGGACTGTTCAGGCTGAGCACCGCTTGCGGCTTGCTGGTTATTGTAGGCTGGAGCTGCTTCG-3	This study
acrB ko-F	5-ATGCCTAATTTCTTTATCGATCGCCCGATTTTTGCGTGGGTGATCGCCATATATGAATATCCTCCTTAG-3	This study
acrB ko-R	5-TCAATGATGATCGACAGTATGGCTGTGCTCGATATCTTCATTCTTGCGGCTGTAGGCTGGAGCTGCTTCG-3	This study
tolC ko-F	5-ATGAAGAAATTGCTCCCCATTCTTATCGGCCTGAGCCTTTCTGGGTTCAGATATGAATATCCTCCTTAG-3	This study
tolC ko-R	5-TCAGTTACGGAAAGGGTTATGACCGTTACTGGTGGTAGTGCGTGCGGATGTGTAGGCTGGAGCTGCTTCG-3	This study
cpxA ko-F	5-ATGATAGGCAGCTTAACCGCGCGCATCTTCGCCATCTTCTGGCTGACGCTATATGAATATCCTCCTTAG-3	This study
cpxA ko-R	5-TTAACTCCGCTTATACAGCGGCAACCAAATCACCAGCCGTAAACCGCCCATGTAGGCTGGAGCTGCTTCG-3	This study
cpxR ko-F	5-ATGAATAAAATCCTGTTAGTTGATGATGACCGAGAGCTGACTTCCCTATTATATGAATATCCTCCTTAG-3	This study
cpxR ko-R	5-TCATGAAGCAGAAACCATCAGATAGCCGCGACCACGCAAGGTTTTAAACCTGTAGGCTGGAGCTGCTTCG-3	This study
soxS ko-F	5-ATGTCCCATCAGAAAATTATTCAGGATCTTATCGCATGGATTGACGAGCAATATGAATATCCTCCTTAG-3	This study
soxS ko-R	5-TTACAGGCGGTGGCGATAATCGCTGGGAGTGCGATCAAACTGCCGACGGATGTAGGCTGGAGCTGCTTCG-3	This study
gshA d-F	5-CTATGGTGCAGTCACGCTATTA-3	This study
gshA d-R	5-GGCATCACTTCTTTGTTCGGCA-3	This study
gshB d-F	5-ATGAAGGACTGAAACTGAATCT-3	This study
gshB d-R	5-CCAGAATCCCTTCAATTTTGAG-3	This study
grxA d-F	5-ATGGCTCAGGCAGCGACAGGGA-3	This study
grxA d-R	5-GCTTGCTATTTTTCATTCTTCC-3	This study
cydD d-F	5-AGTCGGGTATTCATGGTAATGC-3	This study
cydD d-R	5-ACTTACCAGACGTTCAAAATAG-3	This study
gsiA d-F	5-GGCACGGGCGAAGTCTTCATCC-3	This study
gsiA d-R	5-TTATCCAGACCGAACAGCCCCT-3	This study
gsiB d-F	5-GATGTATCTCGGGCAAATTGTT-3	This study
gsiB d-R	5-GCTGGCAATCTCATCGGCAACC-3	This study
gsiAB d-F	5-CTGCGAGCGGGTAGTAATGGAA-3	This study
gsiAB d-R	5-GCTGCTGACGCACCAGTTCTAT-3	This study
gsiC d-F	5-ACTTCCTGGCTCAGGCACTGAA-3	This study
gsiC d-R	5-AAAATGTCACGCCCCAGTGAAT-3	This study
gsiD d-F	5-GAGGGCGAAAGGGGTGAGCGAA-3	This study
gsiD d-R	5-GTTAATGATGAAAAAAAGACCA-3	This study
ggt d-F	5-AGTGCCGTAAGAATCAGAAGTCG-3	This study
ggt d-R	5-CCGCTGGAAGAATATCTACAGGAT-3	This study
acrA d-F	5-GCCCAGCGAGGTGGATGATACCCCC-3	This study
acrA d-R	5-GGTACCCGTGGAGTCACTGTTAGAG-3	This study
acrB d-F	5-GCGGATGACAAAGTGGAAACCC-3	This study
acrB d-R	5-GGTGACACTAATACCAGGATTGCTC-3	This study
tolC d-F	5-ATGTAACGGGCAGGTTGTCTGG-3	This study
tolC d-R	5-CGATGCGTGGCGTATGGATTTT-3	This study
cpxA d-F	5-GCATCTGGGTCAGGTGGTTTCC-3	This study
cpxA d-R	5-TGACGATCAATATCACCGTTTACTCT-3	This study

cpxR d-F	5-TCGAACATATGGCTCTGCGTACTGC-3	This study
cpxR d-R	5-CTGTCCTGGCGGTGCCCACTTATCA-3	This study
soxS d-F	5-GTCGCCAGCGGAATGCCAATAC-3	This study
soxS d-R	5-AACCTTAAATCATTGAATGTCG-3	This study
gapA-RT-F	5-ACTGACTGGTATGGCGTTCC-3	This study
gapA-RT-R	5-GCAGCTTTTTCCAGACGAAC-3	This study
acrA-RT-F	5-TGGCGCGGATGACAAAG-3	This study
acrA-RT-R	5-CCAATAGCCTGGCTTGCAA-3	This study
acrB-RT-F	5-TGGTGACCGCAACGGTACT-3	This study
acrB-RT-R	5-GGCGAACCACCACAAAGAAT-3	This study
tolC-RT-F	5-AAAACGTTGCACCGCAAAC-3	This study
tolC-RT-R	5-GGCGCATAACCATCAGCAAT-3	This study
soxS-RT-F	5-GGTGACGCATCAGACGCTTGG-3	This study
soxS-RT-R	5-CGAGACATAACCCAGGTCCATTGC-3	This study

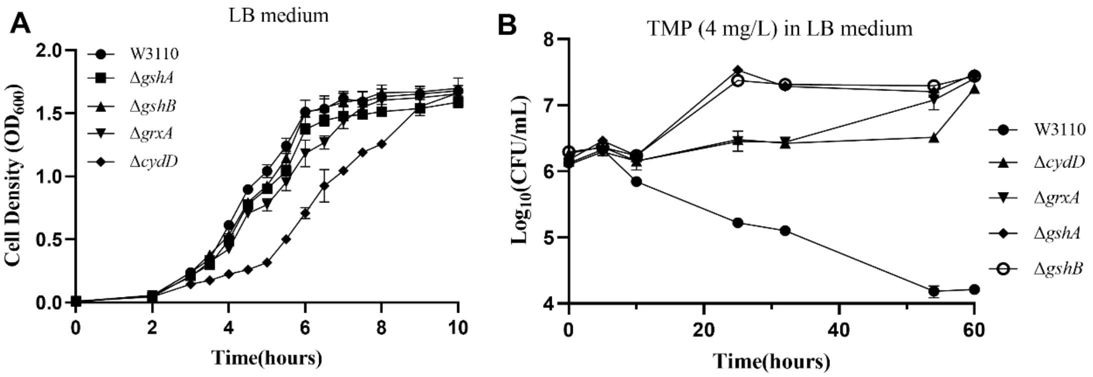

FIG. S1 Growth curves (A) and time-kill curves (B) of different *E. coli* strains in LB medium. Data represent mean ± standard deviation (SD) of the results from three independent experiments.
